# Description of a large measles epidemic in Democratic Republic of Congo, 2010–2013

**DOI:** 10.1186/1752-1505-8-9

**Published:** 2014-07-03

**Authors:** Silvia Mancini, Matthew E Coldiron, Axelle Ronsse, Benoît Kebela Ilunga, Klaudia Porten, Rebecca F Grais

**Affiliations:** 1 Epicentre, 8 Rue Saint Sabin, Paris 75011, France; 2 Médecins Sans Frontières, Via Magenta 5, Rome, Italy; 3 Ministry of Health, Av. De la Justice 39, Gombe I, Kinshasa, Democratic Republic of Congo

**Keywords:** Measles, Epidemic, Infectious Disease, Mass Vaccination, Supplementary immunization activities

## Abstract

**Background:**

Although measles mortality has declined dramatically in Sub-Saharan Africa, measles remains a major public health problem in countries like the Democratic Republic of Congo (DRC). Here, we describe the large measles epidemic that occurred in the Democratic Republic of Congo between 2010 and 2013 using data from the national surveillance system as well as vaccine coverage surveys to provide a snapshot of the epidemiology of measles in DRC.

**Methods:**

Standardized national surveillance data were used to describe measles cases from 2010 to 2013. Attack rates and case fatality ratios were calculated and the temporal and spatial evolution of the epidemic described. Data on laboratory confirmation and vaccination coverage surveys as a part of routine program monitoring are also presented.

**Findings:**

Between week 1 of 2010 and week 45 of 2013, a total of 294,455 cases and 5,045 deaths were reported. The cumulative attack rate (AR) was 0.4%. The Case Fatality Ratio (CFR) was 1.7% among cases reported in health structures through national surveillance. A total of 186,178 cases (63%) were under 5 years old, representing an estimated AR of 1.4% in this age group. Following the first mass vaccination campaigns, weekly reported cases decreased by 21.5%. Results of post-vaccination campaign coverage surveys indicated sub-optimal (under 95%) vaccination coverage among children surveyed.

**Conclusions:**

The data reported here highlight the need to seek additional means to reinforce routine immunization as well as ensure the timely implementation of Supplementary Immunization Activities to prevent large and repeated measles epidemics in DRC. Although reactive campaigns were conducted in response to the epidemic, strategies to ensure that children are vaccinated in the routine system remains the foundation of measles control.

## Background

Globally, a disproportionate number of measles cases and deaths occur in low-income countries with weak health infrastructures [1]. In Sub-Saharan Africa, many countries have not yet introduced a second dose of measles-containing vaccine (MCV) into routine immunization programs. For children in these countries, the current World Health Organization (WHO) and United Nations Children’s Fund (UNICEF) strategy is to deliver the first dose of MCV during routine vaccination programs and the second through regular supplementary immunization activities (SIAs). This strategy reduces the burden of measles [2] and in recent years mortality due to measles has dramatically declined [3]. However, despite the availability of an effective, safe and affordable vaccine [4] and progress in controlling the disease, there were still an estimated 158,000 measles related-deaths in 2011 [5].

The Democratic Republic of Congo (DRC) established the Expanded Program on Immunization (EPI) in 1978. A good barometer for EPI program performance is third-dose vaccination coverage for the Diphtheria-Tetanus-Pertussis vaccine (DTP), which calls for doses at 6, 10 and 14 weeks. Results from the 2007 Demographic Health Survey (DHS) show DTP3 coverage in DRC to be only 45% and the DTP1‒3 drop‒out rate to be 36%, one of the highest rates in the world [6]. More recent results from the Multiple Indicator Cluster Survey (MICS) showed an increase to 61% in country-wide DTP3 coverage although at province level estimates ranged from 27% registered in Maniema to 87% registered in Bandundu Province [7].

**Figure 1:**
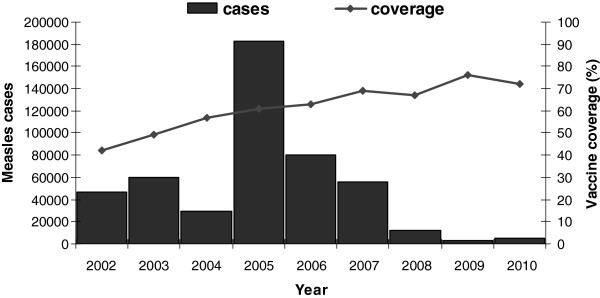
**Evolution of coverage and number of suspected and confirmed measles cases reported from 2002 to 2010, Democratic Republic of Congo.** The bars represent the number of cases reported and the line represents the measles vaccination coverage.

Between 2000 and 2010, first-dose MCV coverage for infants 9–11 months of age increased from 46% to over 70% [8]. Supplementary immunization activities for children 6–59 months were planned at three year intervals to provide either a second dose of measles vaccine or a second opportunity for vaccination to children not receiving their first dose through the routine program. Large measles SIAs were carried out between 2002 and 2004, and then again in 2006–2007. During these campaigns, a total of 30.2 million children were vaccinated. Nonetheless, vaccine coverage remains below the target threshold of 95% (Figure 1). The vast size of the country, its poor infrastructure and transport network, and the weakness of its health system in difficult-to-access areas have resulted in difficulties in improving both routine vaccination and in the implementation of SIAs.

The medical humanitarian organization Médecins Sans Frontières (MSF), present in DRC since 1981, has been an important partner of the Ministry of Health (MOH) in responding to measles epidemics during the past decade. Standard epidemic responses include reinforcing measles surveillance in affected areas, providing free care to reduce measles mortality, and reactive vaccination campaigns in order to stop measles transmission during epidemics. In recent years, MSF has collaborated with the central MOH to improve measles surveillance activities across the country and carried out multiple outbreak response vaccination campaigns. Over the past ten years, measles outbreaks have been reported in several Provinces (Kinshasa, Bas Congo, Maniema, Kasai Oriental) with many reported after significant delay according to Ministry of Health [7]. From 2005 to 2008 the genotype B2 has been identified and circulating in DRC [9].

The current large measles epidemic began in DRC in October 2010. Five provinces (Katanga, Kasaï Oriental, Kasaï Occidental, Sud Kivu, Maniema) were primarily involved, but Equateur and Oriental provinces were also affected [10]. In each of these provinces, SIAs campaigns were scheduled for 2010 but had not yet been conducted when the epidemic started. Financial and logistic reasons were at the origin of this delay, which undoubtedly contributed to increasing the pool of susceptible individuals. Here we present national surveillance data from 2010–2013, the period coinciding with this major measles epidemic.

## Methods

Measles cases in DRC are routinely reported through the integrated disease surveillance (IDS) system, which aims to strengthen surveillance capacity, to better identify disease prevention priorities and to provide epidemiological information for disease control. The IDS functions at several different levels. At the lowest level, information on measles and other reportable diseases is collected on a weekly basis in Health Areas. Information then passes upwards through Health Zones (HZ), Health Districts (HD), and provincial-level ministries of health, which are responsible for transmitting data to the central-level MOH in Kinshasa. An HZ has a variable catchment area, with populations ranging from 26,547 inhabitants in Kowe, Katanga Province, to an estimated 284,187 inhabitants in Ibanda, South Kivu Province.

Following WHO guidelines, in DRC, a suspected measles case is defined as fever >38°C and rash, with at least one of the following: cough, coryza or conjunctivitis. A confirmed measles case is a suspected case with positive IgM antibodies for measles, or a suspected case occurring during an already-confirmed outbreak. A measles death is defined as death from an illness that occurs in a confirmed case of measles within one month of the onset of a rash. All data on cases and deaths were collected on a standard case report form and were routinely transmitted through the IDS system. In the current epidemic, blood specimens of early suspected cases in each HZ (between 5 and 10) were collected and laboratory confirmed by the National Institute of Biomedical Research (INRB) in Kinshasa, where measles and rubella IgM ELISA are performed.

Epidemic definitions are also standardized: a suspected measles epidemic is defined as five or more suspected cases of measles in one HZ within one month and a confirmed measles epidemic is defined as at least three laboratory-confirmed measles cases in a HZ within one month. An epidemic alert was defined as 1 or 2 laboratory-confirmed measles cases in one HZ, or as a HZ without a recent vaccination campaign that neighbored a HZ already in epidemic. Of note, once an epidemic has been declared in an area, serological confirmation is not continued. In the presence of a laboratory-confirmed epidemic, all suspected cases based on clinical criteria are considered confirmed cases.

WHO regional guidelines recommend case-based surveillance for measles [11], but this practice is not yet universal in DRC. Only a limited number of cases were registered with age and vaccination status as aggregate data is presented on the IDS form. Available information included numbers of suspect and confirmed cases by age group, as well as reported mortality. This was collected from the Epidemiologic Surveillance Division of the MOH in Kinshasa.

In addition to surveillance data, we also present information on estimated vaccination coverage from routine monitoring surveys conducted post-vaccination campaign. Between 2010 and 2013, MSF conducted multiple household-based surveys in different areas of DRC, providing local information on vaccination coverage. Results of these surveys and their respective methodologies are reported elsewhere [12,13].

As IDS data is not routinely digitized, data entry was performed in Microsoft Excel. We calculated attack rate (AR), expressed as measles cases divided by the population at risk, and case fatality ratio (CFR), defined as measles deaths divided by the clinically confirmed and suspected measles cases. For the former, the population at risk is a projection based on implied growth rates applied to the 1984 census, the DRC’s most recent.

### Ethical considerations

This descriptive analysis used routine surveillance data collected by the MOH and MSF. Data were not nominative, and authorization for using the data was obtained from the MOH. This data was exempt from review by the MSF Ethical Review Board (ERB) and the national ERB in Kinshasa as routinely collected data were used. During vaccine coverage surveys, verbal informed consent was obtained from each head of household visited [12]. Privacy and confidentiality of patients were ensured by ensuring that no identifying information was recorded.

### Findings

**Figure 2:**
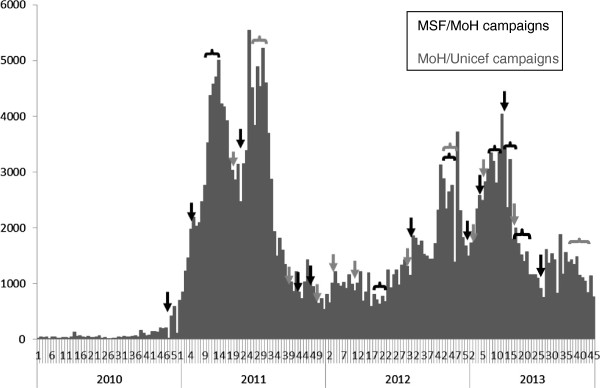
**Number of suspected and confirmed measles cases reported by week, Democratic Republic of Congo, week 1–2010 to week 45–2013.** During this period reactive vaccination campaigns were carried out by Médecins Sans Frontières (MSF) and Ministry of Health (MoH) in collaboration with WHO Afro and Unicef. Black arrows and brackets indicate campaigns conducted by MSF and the MoH. Gray arrows and brackets indicate the campaigns conducted by WHO and Unicef in collaboration with MoH. Brackets indicate that the campaigns were carried out over several weeks.

**Figure 3:**
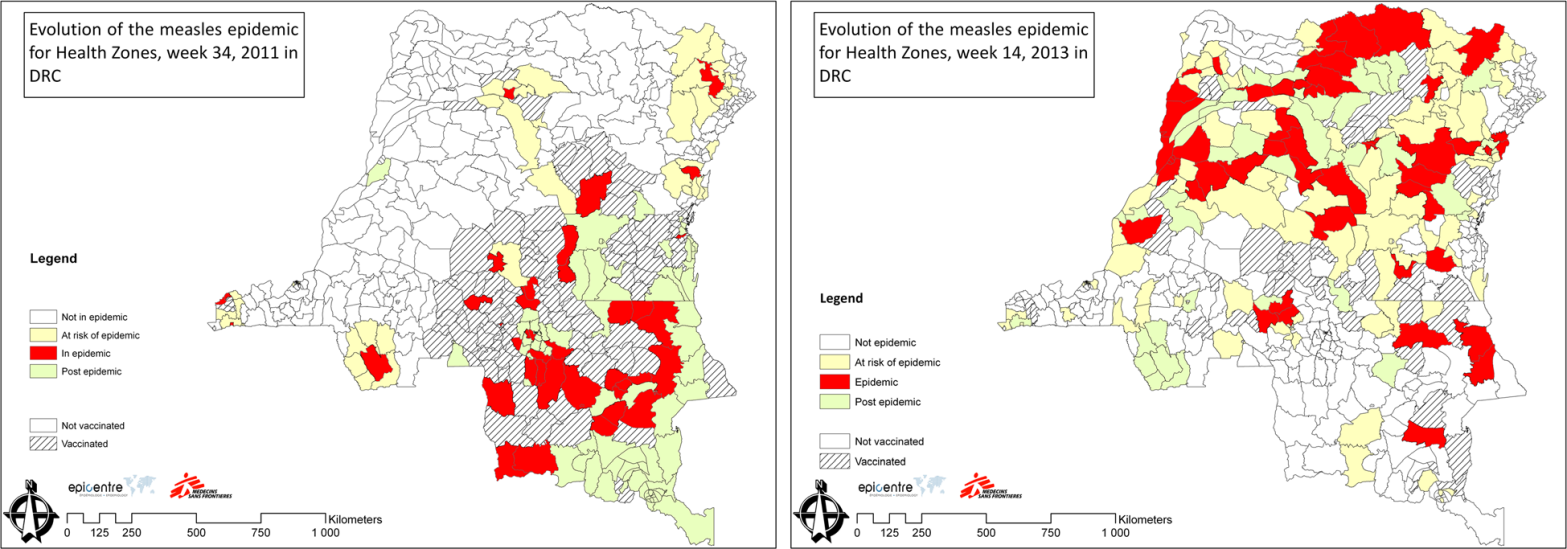
**Health Zones in epidemic and on alert for measles, Democratic Republic of Congo, week 34–2011 and week 14–2013.** We present two points in time to represent the spatial and temporal evolution of the epidemic. White areas represent health zones (HZ) not in epidemic, yellow areas represent the HZs at risk of epidemic, the red areas represent the HZs in epidemic and the green areas the HZs in post epidemic. In the first map, cross-hatched HZ had been vaccinated between October 2010 and August 2011, in the second, cross-hatched HZ had been vaccinated between January 2012 and April 2013.

In April 2010, Kabalo HZ of Katanga Province reported an increase in suspected measles cases. By week 36–2010, two additional HZ (Sakania and Dilolo) reported an increased number of cases. The first samples were confirmed as being measles IgM-positive by ELISA at the INRB during week 38–2010. Since then, measles cases continued to be reported in every province of the country. MSF, in collaboration with the MoH, implemented vaccination campaigns in Sakania and Dilolo during weeks 41 and week 48–2010, respectively.Numbers of reported measles cases by week on a national level between weeks 1–2010 and 45–2013 are presented in Figure 2. During 2011, we note two large peaks, due mostly to cases reported in the Katanga and Kasaï Oriental provinces. During these peaks, over 5,000 measles cases were reported each week. Cases decreased slightly from 5033 to 3920 per week following the first mass vaccination campaigns in weeks 17 and 18–2011 which took place in part of Kasaï Oriental, part of Kasaï Occidental, part of Katanga, in Maniema and in Sud Kivu, then decreased sharply (69%) by week 33–2011 following a second mass vaccination campaign which was extended to the remaining health zones of Kasaï Oriental, Kasaï Occidental and Katanga. The number of reported cases then remained relatively low through early 2012. By the second half of 2012, reported cases began to rise again, this time driven by cases reported in Equateur and Orientale provinces. This uneven geographic distribution of cases is seen in Figure 3, which presents measles data by individual HZ – showing the HZ post-epidemic, in epidemic phase, or on epidemic alert. Two weeks, representative of the peaks of 2011 and 2013, are shown.

Nationwide numbers of reported cases and measles-related deaths by year for the period between weeks 1–2010 and 45–2013 are presented in Table 1. A total of 294,455 cases were reported during this period, over 70% of which occurred in children under 5 years of age. 5,045 measles-related deaths were reported over the same period, representing an overall reported CFR of 1.7%. There was significant yearly variation in the reported CFR, ranging from 1.2% in 2011 to 2.7% in 2012. A total of 2946 cases were laboratory-confirmed between July 2010 and December 2012. A large proportion (29%) of confirmed cases aged between 9 months and 4 years reported never having received a measles vaccination, and 37% of confirmed cases had no available information about prior vaccination status.

**Table 1:** Suspected and confirmed measles cases and deaths reported by year, Democratic Republic of Congo, 2010-2013

**Year**	**Total cases**	**Cases <5 years old, n (% of total cases)**	**Deaths**	**CFR**^**††**^
2010	4 861	2 615 (74.4)	79	1.6%
2011	133 801	101 142 (75.6)	1 646	1.2%
2012	73 844	51 606 (69.9)	2 023	2.7%
2013*	81 949	29 815 (--)^†^	1 297	1.6%
Total	294 455	186 178 (63)	5 045	1.7%

*Only from week 1 to week 45.

^†^This percentage is not calculated as the number of cases aged <5 was not reported between weeks 1 and 12–2013.

^††^CFR: Case Fatality Ratio is the proportion of measles deaths divided by the clinically confirmed cases within a given period time.

**Figure 4:**
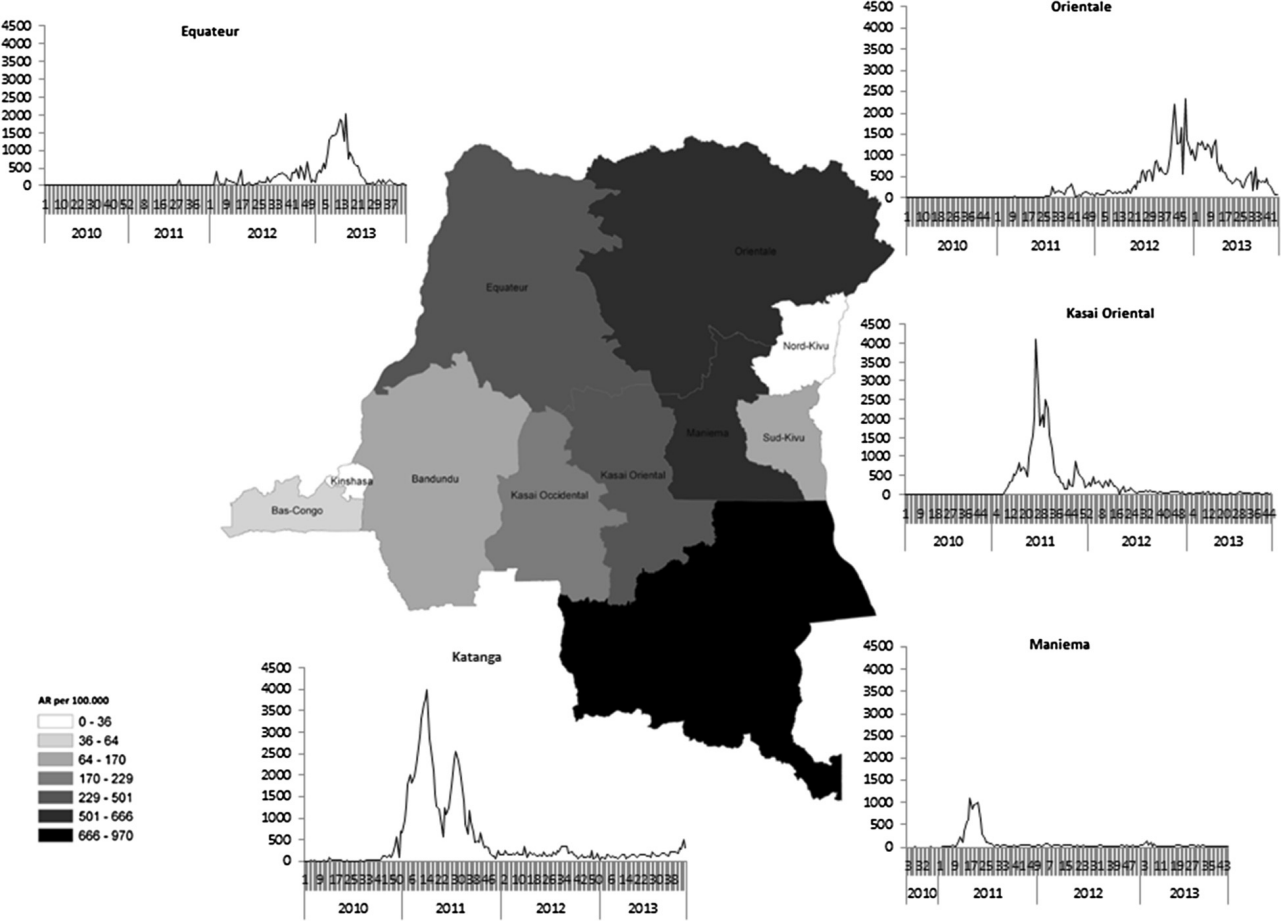
**Measles cumulative attack rate (ARs) per 100 000 inhabitants by province, Democratic Republic of Congo, week 1–2010 through 43–2013.** ARs are presented in gray scale on the map (darker represents higher AR). Weekly incidence is presented for the most affected provinces (x-axis represents epidemiologic week by year, y-axis represents number of incident cases reported).

The overall cumulative AR for this epidemic was 0.4%, and 1.4% among children under 5 years. Cumulative AR by province is shown in Table 2. This information is also presented in Figure 4, which combines province-level attack rates with province-level epidemic curves.

**Table 2:** Cumulative measles attack rates by province, Democratic Republic of Congo, 2010-2013

**Provinces**	Total population*	**Measles cases 2010-2013**	**AR%**
Bandundu	7 714 915	13 136	0.17%
Bas Congo	3 334 201	2 143	0.06%
Equateur	8 629 816	34 172	0.40%
Kasaï Occidental	7 216 209	16 595	0.23%
Kasaï Oriental	9 136 786	47 899	0.52%
Katanga	9 707 496	94 181	0.97%
Kinshasa	7 092 711	2 531	0.04%
Maniema	2 009 182	12 312	0.61%
Nord Kivu	6 347 169	2 229	0.04%
Orientale	9 487 106	63 272	0.67%
Sud Kivu	4 864 044	5 985	0.12%
Total	75 539 635	294 455	0.39%

*Average projected population over the period 2010-2013.

During this period, reactive vaccination campaigns were carried out in 190 out of 516 HZ and SIAs were organized in 378 HZ (some HZ received reactive campaigns later followed by SIAs). Most targeted children aged 6 months – 15 years, others targeted children aged 6 months – 10 years and others 6 months – 5 years.

Results from vaccine coverage surveys carried out by MSF are presented in Table 3. All of these surveys took place in areas where reactive vaccination campaigns occurred, thus all were in HZs with measles epidemics. It is important to note the great variation in pre-campaign coverage, a surrogate marker for prior vaccination opportunities, e.g. EPI, SIAs.

**Table 3:** **Results of routine vaccine coverage surveys, DRC, 2011–2013**^**1**^

**Province**	**Health district**	**EPI VC before mass campaign**		**VC after mass campaign**	
		**By card and oral**		**By card and oral**	
Kasaï Occidental	Tshikapa	54.4%	[43.5-65.3]	97%	[94.8-99.3]
Orientale	Watsa	57.3%	[50.4-64.1]	99.2%	[98.5-100]
Katanga	Sakania	69.1%	[58.7-79.5]	95.3%	[92.3-98.4]
Katanga	Kasenga	86.2%	[80.5-90.4]	93.7%	[91–95.7]
Katanga	Kapolowe	83.1%	[74–89.4]	97%	[88–99.3]
Kasaï Oriental	Mwene Ditu	70.5%	[63.1-77.8]	98.8%	[97.6-100]
Equateur	Yambuku	39.70%	[28.2-51.4]	95.4%	[92.1-98.6]
Orientale	Yaleko	39%	[31.2-47.3]	97.6%	[96.4-99.9]
Orientale	Yalimbongo	34.4%	[23.1-47.4]	98.2%	[96.5-99.9]
Orientale	Yahuma	44%	[36–52]	95%	[93–98]
Orientale	Yahisuli	49%	[39–58]	97%	[95–99]

^1^Data from Katanga Province were first published in Grout L, Minetti A, Hurtado N, François G, Fermon F, Chatelain A, Harczi G, Ilunga Ngoie J, N’Goran A, Luquero J F, Grais R F, Porten K: **Measles in Democratic Republic of Congo: an outbreak description from Katanga, 2010–2011.** BMC Infectious Diseases, 2013. 13 (1): p.232.

The results of household-based surveys in specific HZ during this period show pre-outbreak response vaccination coverage to be heterogeneous (Table 3). In some HZ, the post-campaign coverage increased up to 98%. This variability is corroborated by the 2007 Demographic Health Survey, which showed varying levels of coverage between provinces. Maniema and Equateur had the lowest vaccination coverage (10% and 15% respectively), while Nord Kivu (67%), Bas Congo (60%) and Kinshasa (58%) had the highest rates.

## Discussion

A lengthy measles epidemic has been occurring in DRC since 2010, and was still ongoing at the end of 2013. To interrupt endemic transmission of measles, mathematical models indicate that 93%-95% of a population must be immune [14], a distant threshold in DRC, where a lack of access to care, political instability, and poor physical infrastructure have led to low routine vaccination coverage and continuing measles endemicity. This endemicity is also reflected in the high proportion of cases seen among children under 5. Given that MCV coverage has been quite low for many years, it is likely that older children and adults have been protected against measles via naturally-acquired immunity after prior exposure to measles.This prolonged epidemic has spread across the country over a period of almost 3 years. The data presented at the level of HZs (Figure 3) show a slow progression of the epidemic across the country which confirms subjective impressions of the epidemic’s spread. Nonetheless, it is important to note that some HZ in the north were in epidemic phase in 2011, and that some HZ in the south were in epidemic phase in 2013, further illustrating measles endemicity throughout DRC.

Considering the steep increase in measles cases in Eastern and Southern Africa between 2009 and 2010, we cannot exclude the possibility that this epidemic might have resulted also from multiple introductions from neighboring countries. From 2005 to 2008, genotype B2 was identified as circulating in DRC [9], but molecular information from the epidemic in 2010–2013 showed the circulation of both genotype B2 and B3, the same genotypes reported in recent epidemics in Angola and Zambia [14]. The HZ of Sakania, adjacent to Zambia, reported an increase in cases at the end of 2010, and the HZ of Dilolo (Katanga) and Kamonia (Kasai Occidental), both bordering Angola, reported increase in cases between weeks 36 and 46–2010.

Nonetheless, several caveats about the data should be highlighted.

First, the attack rates presented are calculated using population estimates based on the 1984 census. For many reasons, these population estimates, based on an annual growth of 3%, are likely inaccurate. Use of population projections is a known weakness and in countries without accurate population data, and contributes to difficulties in assessing coverage. This is particularly evident when examining administrative vaccination coverage (number of doses administered divided by the presumed target population), where rates of over 100% are often reported [15]. However, they are the standard figures used in the DRC, and remain the most rational option until a new census is conducted.

Secondly, the case fatality ratios should also be interpreted with caution. They were low compared with other African settings [16-19]. In the DRC, where access to care is limited, the low CFR is likely due to an underreporting of deaths, many of which happen outside of health structures. At the same time, measles cases may have been overestimated using the clinically confirmed case definition, as some provinces had concurrent rubella epidemics.

Finally, the surveillance system in DRC is still a work in progress: line lists were not completed in most districts, and only aggregate age information was recorded. The completeness of data is weak, and the sensitivity of the surveillance system has not been formally evaluated [14].

In summary, in a country the size of Western Europe, case reporting for measles and many other diseases is incomplete, varying greatly by province. Therefore epidemiological analysis did not fully represent all measles cases in the country.

Despite the variation in baseline MCV coverage, it is important to note that all of the areas experiencing epidemics had a pre-epidemic MCV coverage below 95%. The end result is an epidemic that has caused almost 300,000 cases and over 5,000 deaths. The heterogeneity of the epidemic is consistent with the heterogeneity of vaccine coverage in the country. The Demographic Health Survey of 2007 estimated nationwide MCV1 coverage at 63%, but vaccination coverage varied greatly among the provinces. Katanga and Oriental provinces (both greatly affected in the current epidemic) had a coverage respectively of 51% and 49%. In contrast, the highest levels of vaccination coverage were in North Kivu (85%), Bas Congo (88%) and Kinshasa (91%), the least-affected provinces in this epidemic. In 2012, a vaccination coverage survey conducted by the Public Health University of Kinshasa, confirmed a similar situation [20].

WHO guidelines for outbreak response in measles mortality reduction settings like the DRC recommend non-selective vaccination campaigns to control epidemics [11]. Nonetheless, in a country the size of the DRC with its problems of inaccessibility, the sheer logistical burden presented by the prospect of carrying out many campaigns at the same time is daunting. Many of the reactive vaccination campaigns conducted during this epidemic were carried out relatively late in the course of the epidemic in the given areas. This likely had a positive impact on the epidemic curve [21], but the impact would likely have been greater with an earlier response [22,23]. Some of the campaigns were conducted in some of the most affected districts but not always in neighboring districts where the epidemic could have spread. This leads further credence to the importance of context-specific approaches when planning mass vaccination responses to epidemics [24].

In health zones where MSF was implementing the reactive campaigns, the health surveillance system was strengthened with additional data collection, retrospective review of health registers and monitoring of data completeness. This more complete data revealed that cases were concentrated in children under 5 years old and the AR was lower for children older than 10 years – a finding that led to the decision to vaccinate children between 6 months and 10 years old in some settings. This approach could be applied in future responses to ensure the appropriate targeting of children, especially in difficult to reach areas with limited resources [25].

Ensuring a first dose in the routine program, followed by regular SIAs every two or three years, is the most efficient, and surely the most cost-effective way to increase measles vaccine coverage [26-29]. In the meantime, case-based measles surveillance should be strengthened and prompt outbreak investigations should be used to complement vaccination coverage information to identify gaps in population immunity.

## Conclusions

Measles vaccination coverage in the DRC has improved in recent years but is still far below protective levels, leading to this large epidemic which affected mainly children under 5 years old. Reactive vaccination campaigns have been an important part of the response to this epidemic and have helped to increase vaccination coverage to desirable levels but they cannot be considered a sustainable measles control strategy over the long-term. Achieving a high coverage through a reinforcement of the routine immunization program and effective SIAs is the best way to prevent measles epidemics from occurring. Routine vaccination activities, as well as the nascent surveillance system, must be strengthened with a special focus on the case-based system. Detailed outbreak investigations and collection of additional data (compilation of line-lists) are recommended to describe the epidemiology and the age distribution of the epidemic, to guide rapid and effective reactive immunization campaigns and to target the most appropriate age groups. Population-based coverage surveys should be implemented after SIA activities to determine the susceptibility profile of the population and to identify areas of low coverage to better prioritize and efficiently use of resources.

## Abbreviations

AR: Attack Rate; CFR: Case Fatality Rate; DRC: Democratic Republic of Congo; ELISA: Enzyme-linked immunosorbent assay; EPI: Expanded Program on Immunization; HD: Health District; HZ: Health Zone; IDS: Integrated Disease Surveillance; IgM: Immunoglobulin M; INRB: Institut National de Recherche Biomédicale (National Institute of Biomedical Research); MCV: Measles Containing Vaccines; MoH: Ministry of Health; MSF: Médecins Sans Frontières/Doctors Without Borders; ORI: Outbreak Response Immunization; SIA: Supplementary Immunization Activities; UNICEF: United Nations Children’s Fund; WHO: World Health Organization.

## Competing interests

None of the authors has any competing interests, financial or otherwise.

## Authors’ contributions

SM implemented the study, collated the information from the field, analyzed the data and drafted the manuscript. MC participated in the critical revision and helped to draft the manuscript. AR participated in the design of the study and to collect the data. BKI and RFG participated in the interpretation of the results and with critical revision of the manuscript. KP participated in the design and coordination of the study and helped to draft the manuscript. All authors read and approved the final manuscript.
